# Insanity Not a Result of the Separate System of Imprisonment

**Published:** 1848-05

**Authors:** 


*Insanity not a result of the separate system of imprisonment.*—From a report on the Pentonville Prison, it appears that in the first year (1843) of 332 convicts, the daily average in the prison, three became affected with insanity. In 1844, when the daily average was 456, no case occurred. There was one in each of the two following years,—when the daily averages were respectively 445 and 423. In 1843, the cases were in proportion of 9.03 per 1000. During the whole period (four years and a quarter) since the prison was opened, the proportion of cases to the daily average of prisoners has been that of 2.29 per 1000 annually. From the end of 1843 to the present time, the annual proportion has been no more than 1.48 per 1000. If we compare the amount of mental disease in the army, where it can be accurately ascertained, we find that per 1000 it is nearly 1. among the Dragoon-guards in the home service, 1.43 in the Ionian islands, 1.33 in Canada, and 1.41 in Gibraltar. The statement, therefore, that the separate system tends to produce insanity has no foundation in fact. *London Med. Gaz.*

